# Astrocyte-specific overexpression of Nrf2 protects against optic tract damage and behavioural alterations in a mouse model of cerebral hypoperfusion

**DOI:** 10.1038/s41598-018-30675-4

**Published:** 2018-08-22

**Authors:** Emma Sigfridsson, Martina Marangoni, Jeffrey A. Johnson, Giles E. Hardingham, Jill H. Fowler, Karen Horsburgh

**Affiliations:** 1 Centre for Discovery Brain Sciences, University of Edinburgh, Edinburgh, UK; 2 Division of Pharmaceutical Sciences, University of Wisconsin, Madison, USA; 3 Molecular and Environmental Toxicology Center, University of Wisconsin, Madison, USA; 4 Center for Neuroscience, University of Wisconsin, Madison, USA; 5 Waisman Center, University of Wisconsin, Madison, USA; 6 Edinburgh Medical School, University of Edinburgh, Edinburgh, UK; 7 The UK Dementia Research Institute, University of Edinburgh, Edinburgh, UK; 8 Present Address: Department of Health Sciences, University of Florence, Florence, Italy

## Abstract

Mouse models have shown that cerebral hypoperfusion causes white matter disruption and memory impairment relevant to the study of vascular cognitive impairment and dementia. The associated mechanisms include inflammation and oxidative stress are proposed to drive disruption of myelinated axons within hypoperfused white matter. The aim of this study was to determine if increased endogenous anti-oxidant and anti-inflammatory signalling in astrocytes was protective in a model of mild cerebral hypoperfusion. Transgenically altered mice overexpressing the transcription factor Nrf2 (GFAP-Nrf2) and wild type littermates were subjected to bilateral carotid artery stenosis or sham surgery. Behavioural alterations were assessed using the radial arm maze and tissue was collected for pathology and transcriptome analysis six weeks post-surgery. GFAP-Nrf2 mice showed less pronounced behavioural impairments compared to wild types following hypoperfusion, paralleled by reduced optic tract white matter disruption and astrogliosis. There was no effect of hypoperfusion on anti-oxidant gene alterations albeit the levels were increased in GFAP-Nrf2 mice. Instead, pro-inflammatory gene expression was determined to be significantly upregulated in the optic tract of hypoperfused wild type mice but differentially affected in GFAP-Nrf2 mice. In particular, complement components (C4 and C1q) were increased in wild type hypoperfused mice but expressed at levels similar to controls in hypoperfused GFAP-Nrf2 mice. This study provides evidence that overexpression of Nrf2 in astrocytes exerts beneficial effects through repression of inflammation and supports the potential use of Nrf2-activators in the amelioration of cerebrovascular-related inflammation and white matter degeneration.

## Introduction

Vascular cognitive impairment (VCI) is a spectrum of mild cognitive impairment to vascular dementia and is influenced by risk factors including age, hypertension and atherosclerosis. The most common form of VCI is small vessel disease which is predominantly associated with white matter changes that can be detected as hyperintense signals on FLAIR or T2-weighted magnetic resonance images^1,2^. White matter changes correlate with cognitive decline^3,4^ and are closely related to reduced cerebral perfusion^5,6^. The extent and presence of white matter changes can predict development of dementia in patients with mild cognitive impairment^7,8^. Thus, understanding pathophysiology of white matter changes has important implications in the treatment of dementia.

Models of cerebral hypoperfusion have been important in providing mechanistic insight into the pathophysiology of VCI; the best characterised is the bilateral carotid artery stenosis (BCAS) model in mice^9,10^. We and others have demonstrated that cerebral hypoperfusion in mice disrupts myelinated axons within the white matter^11,12^ causing impaired spatial working memory^11,13,14^. Increased inflammatory cells, particularly microglia, often parallel hypoperfusion-induced white matter damage^9,15,16^. Furthermore, there appears to be a close link between damage to white matter, microgliosis and white matter function in response to mild^17^ and severe cerebral hypoperfusion^18^. Our work demonstrated that the use of a broad spectrum anti-inflammatory drug, minocycline, markedly attenuates microgliosis and improves white matter function in a mouse model of cerebral hypoperfusion^17^, and has also identified activation of pro-inflammatory genes within days in hypoperfused white matter^12^. Inflammation is often accompanied by indices of oxidative stress which is also proposed as a key contributor to pathology following cerebral hypoperfusion (reviewed by^19,20^). Increased levels of the reactive species superoxide, the superoxide-producing enzyme NADPH oxidase and oxidative damage to lipids, proteins and nucleic acids is found in hypoperfused white matter^21–23^. Reduced anti-oxidant and detoxifying enzymes and/or dysfunctional mitochondria are suggested underlying mechanisms^24–26^.

Deficiency of the transcription factor nuclear factor erythroid 2-related factor (Nrf2), a master regulator of endogenous cytoprotective anti-oxidant and anti-inflammatory gene pathways, is associated with white matter damage. Nrf2 knockout mice exhibit myelin pathology characterised by myelin unwinding, lipid peroxidation of the myelin sheath, and increased astrocytosis^27^, as well as reduced functional recovery and remyelination following sciatic nerve crush and experimental autoimmune encephalitis (EAE)^28,29^. In contrast, activation of Nrf2 using dimethyl fumarate (DMF) has been shown to prevent myelin damage and astrocyte activation in EAE^30^, and DMF has since been approved for the treatment of relapsing-remitting multiple sclerosis (reviewed by^31^). Recently we have also shown that treatment with DMF, in a severe model of cerebral hypoperfusion, ameliorates white matter functional impairment and microgliosis^18^. Nrf2 expression has been shown to be several fold higher in astrocytes compared to neurons^32^ which have been shown to repress Nrf2 expression developmentally as redox-sensitive signalling pathways are important for proper maturation, and instead neurons rely on astrocytic support to prevent oxidative damage^33^. Oligodendrocytes, also highly metabolically active cells, receive anti-oxidant support from astrocytes as well^34^, which may explain their comparably higher levels of Nrf2 expression. Studies using GFAP-Nrf2 mice in models of familial amytrophic lateral sclerosis, Huntington’s and Parkinson’s disease find increased production of glutathione and/or glutathione-related genes^35–38^, similarly seen in models of cerebral ischaemia^39,40^, all of which were associated with favourable outcomes.

Since astrocyte specific overexpression of Nrf2 has been shown to confer white matter protection in a number of disease models, we wished to build on this work to interrogate the effects of increased expression of astrocytic Nrf2 on white matter vulnerability and behavioural outcomes which are impaired in response to cerebral hypoperfusion. The novelty of the present study was to utlise a cell specific and genetic approach to investigate the putative protective effects of astrocytic Nrf2, unlike previous studies which have mainly used pharmacological approaches to indirectly assess the effects of Nrf2 in hypoperfusion models^41,42^. We hypothesised that overexpression of astrocytic Nrf2 would alleviate hypoperfusion induced cognitive impairment by reducing white matter disruption and inflammation, through glutathione-related mechanisms.

## Results

### Cortical cerebral blood flow is significantly reduced following bilateral carotid artery stenosis but is not influenced by astrocytic expression of Nrf2

**Figure 1:**
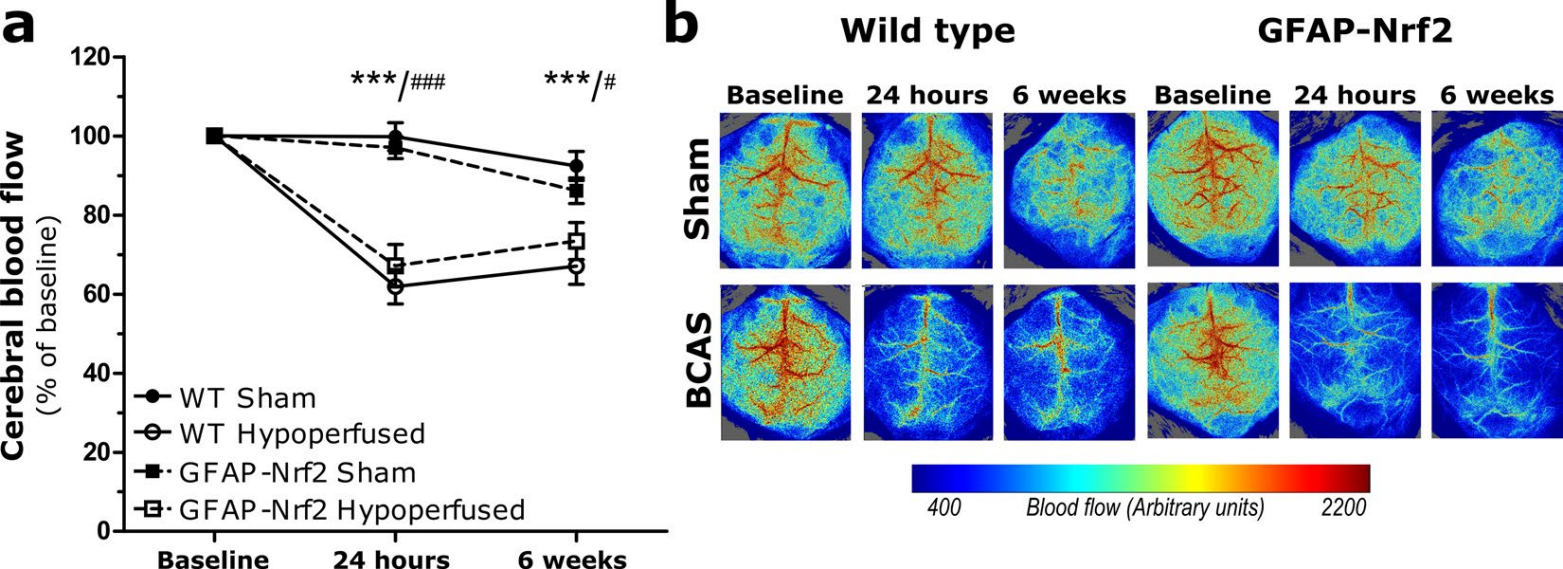
Resting cortical cerebral blood flow (CBF) is significantly reduced following bilateral carotid artery stenosis in wild type and GFAP-Nrf2 mice. (**a**) Resting CBF was significantly reduced by 30–40% from baseline 24 hours after bilateral carotid artery stenosis (BCAS) surgery and remained significantly reduced at 6 weeks for both groups. Effect of time F_(2,64)_ = 57.02, p < 0.0001, effect of surgery F_(1,32)_ = 55.20, p < 0.0001, no significant effect of genotype. (**b**) Representative images of laser speckle flowmetry at baseline and at 24 hr and 6weeks post-surgery. Repeated measures ANOVA with Bonferroni adjustment for post hoc analysis. Mean ± SEM. WT/GFAP-Nrf2 ***/^###^p < 0.001, GFAP-Nrf2 ^#^p = 0.03. n = 8–10 per group.

Cortical cerebral blood flow (CBF) was evaluated user laser speckle imaging at baseline (before surgery) and then at 24 hours and 6 weeks after surgery to assess the temporal response to carotid artery stenosis and to determine if there was a difference between wild type and GFAP-Nrf2 mice (Fig. 1). CBF data for each animal was calculated as a percentage of its baseline CBF. Overall, there was a significant effect of time (F_(2,64)_ = 57.02, p < 0.0001) and bilateral carotid artery stenosis surgery (F_(1,32)_ = 55.20, p < 0.0001) and a significant interaction between time and surgery (F_(2,64)_ = 32.63, p < 0.0001). Post hoc analysis indicated that CBF was significantly reduced in wild type and GFAP-Nrf2 hypoperfused mice from their corresponding sham controls at 24 hours (p < 0.001) and 6 weeks (WT; p < 0.001, GFAP-Nrf2; p = 0.03). No overall effect of genotype was detected (F_(1,32)_ = 0.03, p = 0.86), indicating that overexpression of Nrf2 does not affect resting cortical cerebral blood flow in sham or hypoperfused mice (Fig. 1a).

### Cerebral hypoperfusion causes a behavioural impairment in wild type mice which is less pronounced in GFAP-Nrf2 mice

**Figure 2:**
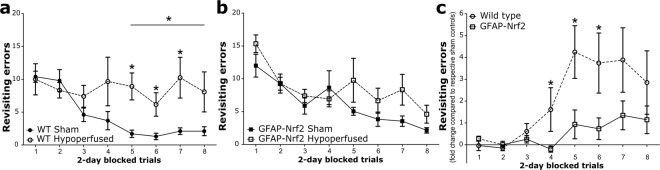
Cerebral hypoperfusion causes an impairment in behavioural performance in wild type mice which is less pronounced in GFAP-Nrf2 mice. Spatial behaviour was assessed by the radial arm maze as revisiting errors over consecutive trials. (**a**) Wild type (WT) hypoperfused animals committed significantly more revisiting errors than WT shams during blocks 5–8 (F_(1,9)_ = 6.56, p = 0.03). (**b**) GFAP-Nrf2 hypoperfused animals did not make significantly more revisiting errors than the GFAP-Nrf2 shams during blocks 5–8 (F_(1,17)_ = 3.81, p = 0.07) (**c**) Revisiting error fold difference compared to respective sham controls was used to investigate genotype effect and analysis revealed a significantly higher fold difference in wild type mice (F_(1,13)_ = 4.79, p = 0.048). Mean ± SEM. Repeated measures ANOVA with Bonferroni adjustment for post hoc analysis. *p < 0.05. n = 5–10 per group.

Behaviour was assessed using an 8-arm radial arm maze which has previously been shown to be sensitive to hypoperfusion-induced disruption of frontocortical circuitry^11,13^. Sham wild type and GFAP-Nrf2 transgenic mice had different baseline performances and different patterns of learning over the trial blocks 1–8 (Fig. S1), and thus revisiting errors were analysed separately for these groups to investigate the effect of hypoperfusion over trial blocks 1–4 and 5–8. There was no significant difference in revisiting errors between wild type sham and hypoperfused mice in trials 1–4 (F_(1,9)_ = 1.51, p = 0.25) but in trials 5–8 wild type hypoperfused mice made significantly more revisiting errors than the sham controls (F_(1,9)_ = 6.56, p = 0.03) (Fig. 2a). Post hoc analysis found significant differences between wild type sham and hypoperfused animals at block 5, 6 and 7 (p = 0.01, 0.04, 0.04 respectively). In contrast, GFAP-Nrf2 sham animals were not significantly different from hypoperfused mice in trials 1–4 (F_(1,17)_ = 0.33, p = 0.57) and in trials 5–8 (F_(1,17)_ = 3.81, p = 0.07) (Fig. 2b). There was an overall significant effect of trial for both wild type (F_(2.9,25.9)_ = 3.39, p = 0.04) and GFAP-Nrf2 mice (F_(4,68.3)_ = 9.47, p < 0.0001) (Fig. 2a,b), indicative of learning.

To investigate whether there may be genotype differences between the groups, the fold difference of revisiting errors of hypoperfused groups was calculated to respective sham controls and then compared between wild type and GFAP-Nrf2 mice. There was an overall genotype effect (F_(1,13)_ = 4.79, p = 0.048) (Fig. 2c), a significant effect of trial (F_(2.5,32.4)_ = 8.70, p < 0.0001) and a significant interaction between trial and genotype (F_(2.5,32.4)_ = 3.36, p = 0.04). Post hoc analysis identified significant differences in the fold change of revisiting errors between wild type and GFAP-Nrf2 groups at blocks 4, 5 and 6 (p = 0.046, 0.02, 0.03 respectively). Thus overall the data suggest a modest protective effect of Nrf2 overexpression on behavioural impairment following cerebral hypoperfusion.

### Loss of optic tract white matter integrity caused by cerebral hypoperfusion is less severe in GFAP-Nrf2 mice

Behavioural performance in the radial arm maze is dependent on the integrity of myelinated axons within white matter for efficient communication. The corpus callosum, internal capsule and optic tract were immunostained for myelin associated glycoprotein (MAG) present at the axon-glial interface. The density of MAG+ immunostaining was quantified as percentage area. When the axon-glial interface is disrupted, there is an accumulation of MAG + debris indicating a loss of white matter integrity^11^.

**Figure 3:**
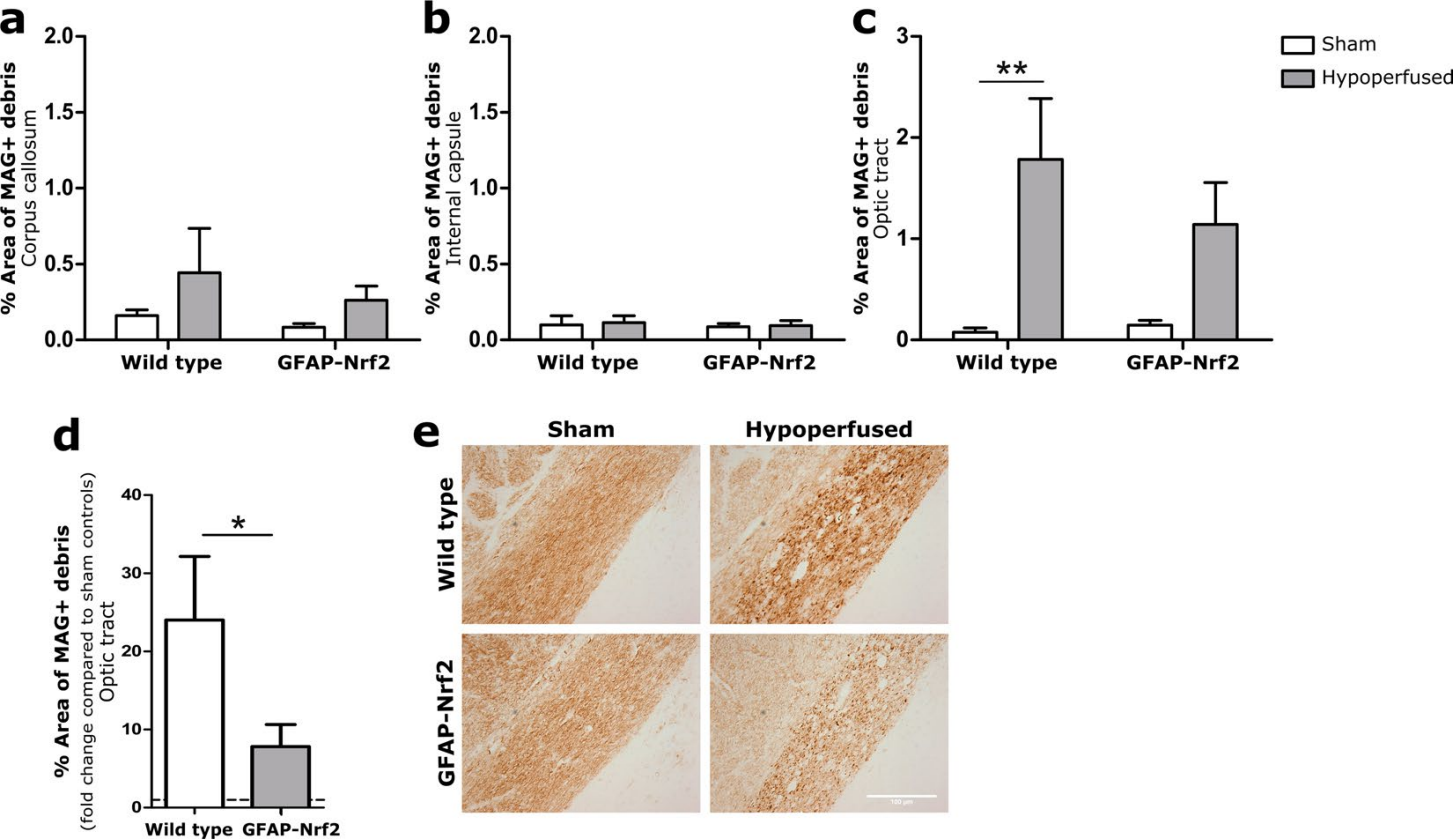
Loss of white matter integrity in response to hypoperfusion. (**a**) There was no significant loss of white matter integrity between groups in the corpus callosum, (**b**) nor in the internal capsule (**c**) but there was a significant effect of cerebral hypoperfusion in the optic tract (F_(1,31)_ = 13.57, p = 0.001). (**d**) Fold change of white matter integrity loss compared to respective sham controls in the optic tract showed significantly more loss in wild type animals (p = 0.03). Dashed line indicates average sham level. (**e**) Representative images of MAG+ staining in the optic tract. Scale bar 100 µm. Mean ± SEM. (**a**–**c**) Two-way ANOVA with Bonferroni adjustment for post hoc analysis. (**d**) Unpaired one-tailed t-test. *p < 0.05, ** p < 0.01. n = 8–10 per group.

There was no significant loss of white matter integrity as a result of hypoperfusion in the corpus callosum (F_(1,32)_ = 2.63, p = 0.12) or the internal capsule (F_(1,32)_ = 0.08, p = 0.78), and no effect of genotype (F_(1,32)_ = 0.82, p = 0.37; F_(1,32)_ = 0.17, p = 0.68 respectively) (Fig. 3a,b). However, there was a significant effect of hypoperfusion in the optic tract (F_(1,31)_ = 13.57, p = 0.001) albeit no significant effect of genotype (F_(1,32)_ = 0.6, p = 0.44). Post hoc analysis of the effect of hypoperfusion revealed a significant loss of white matter integrity in wild type hypoperfused mice compared to sham controls (p = 0.004), but the difference between hypoperfused GFAP-Nrf2 mice and sham controls narrowly missed accepted levels of statistical significance (p = 0.054) (Fig. 3c).

To investigate whether the magnitude of white matter integrity loss differs between genotypes, the fold difference of MAG+ debris compared to controls in the optic tract was calculated and compared between wild type and GFAP-Nrf2 mice (Fig. 3d). The fold difference was significantly higher in wild type mice (p = 0.03) (Fig. 3d), suggesting a modest protective effect of Nrf2 overexpression on white matter integrity following hypoperfusion.

### Cerebral hypoperfusion induces micro- and astrogliosis in the optic tract, and astrogliosis is ameliorated by Nrf2-overexpression

Previously it has been shown that inflammation is a key driver of white matter disruption in cerebral hypoperfusion^17^. To assess the microglial/macrophage inflammatory response, Iba1 immunostaining was used and microglia/macrophages were quantified as Iba1+ percentage area in the main white matter tracts; corpus callosum, internal capsule and optic tract. Glial fibrillary acidic protein (GFAP) immunostaining was utilised to assess the astroglial inflammatory response; quantified as GFAP+ percentage area in the corpus callosum, internal capsule and optic tract.

**Figure 4:**
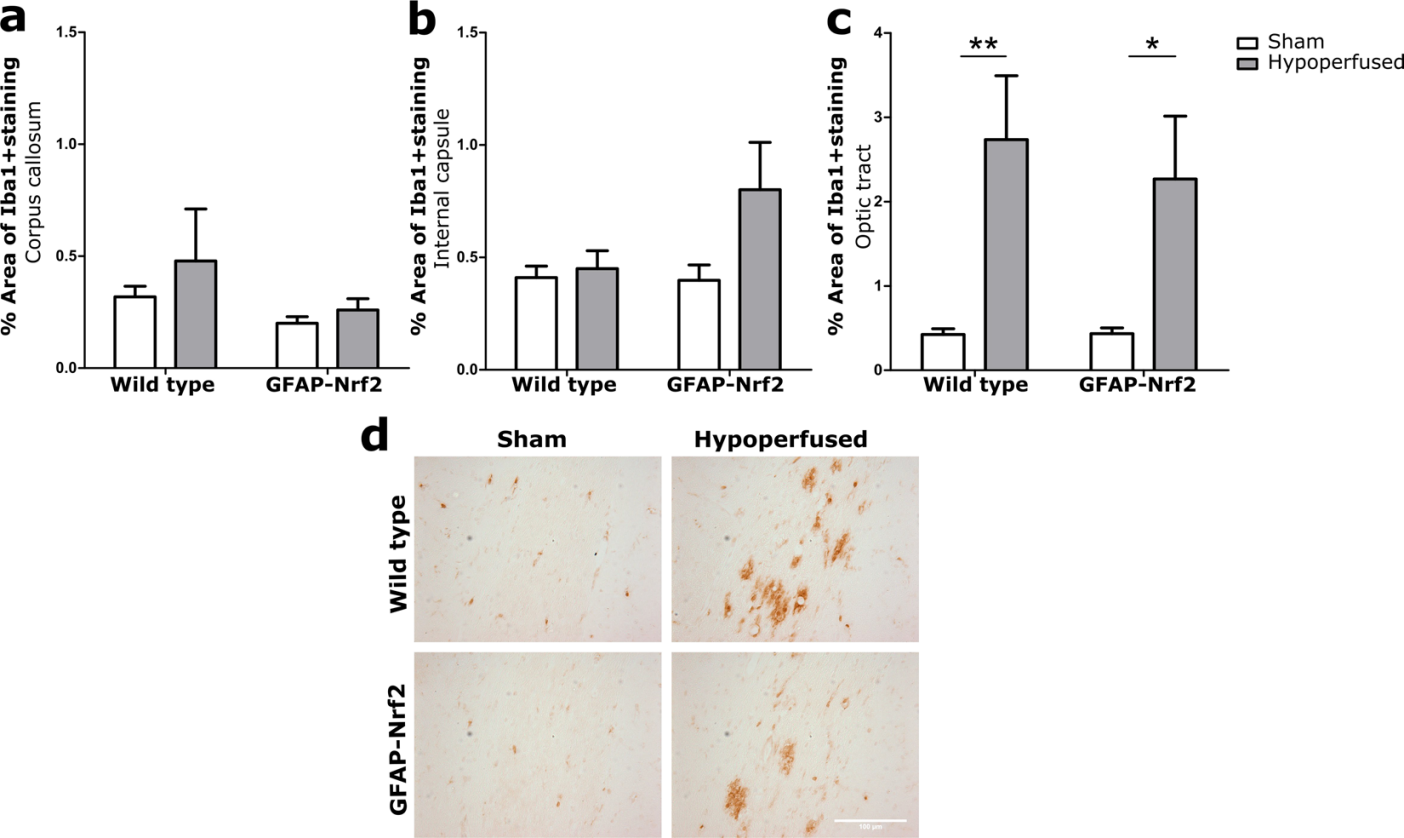
Increased microglia/macrophages in response to hypoperfusion. (**a**) % Area of Iba1+ staining in the corpus callosum and (**b**) internal capsule was unchanged following cerebral hypoperfusion. (**c**) % Area of Iba1+ staining in the optic tract was significantly altered by cerebral hypoperfusion (F_(1,32)_ = 14.74, p = 0.001), with no effect of genotype. (**d**) Representative images of Iba1+ staining in the optic tract. Scale bar 100 µm. Mean ± SEM. Two-way ANOVA with Bonferroni adjustment for post hoc analysis. *p < 0.05, ** p < 0.01. n = 8–10 per group.

Cerebral hypoperfusion had a significant effect on Iba1 expression in the optic tract (F_(1,32)_ = 14.74, p = 0.001) (Fig. 4c), but not in the corpus callosum or the internal capsule (F_(1,32)_ = 2.41, p = 0.13, F_(1,32)_ = 2.75, p = 0.11 respectively) (Fig. 4a,b). Post hoc analysis revealed significantly greater Iba1+ percentage area in both wild type and GFAP-Nrf2 hypoperfused groups compared to their controls (p = 0.007, p = 0.02, respectively). There was no effect of genotype in any region (CC-F_(1,32)_ = 3.12, p = 0.09; IC-F_(1,32)_ = 2.23, p = 0.14; OT-F_(1,32)_ = 0.18, p = 0.68).

**Figure 5:**
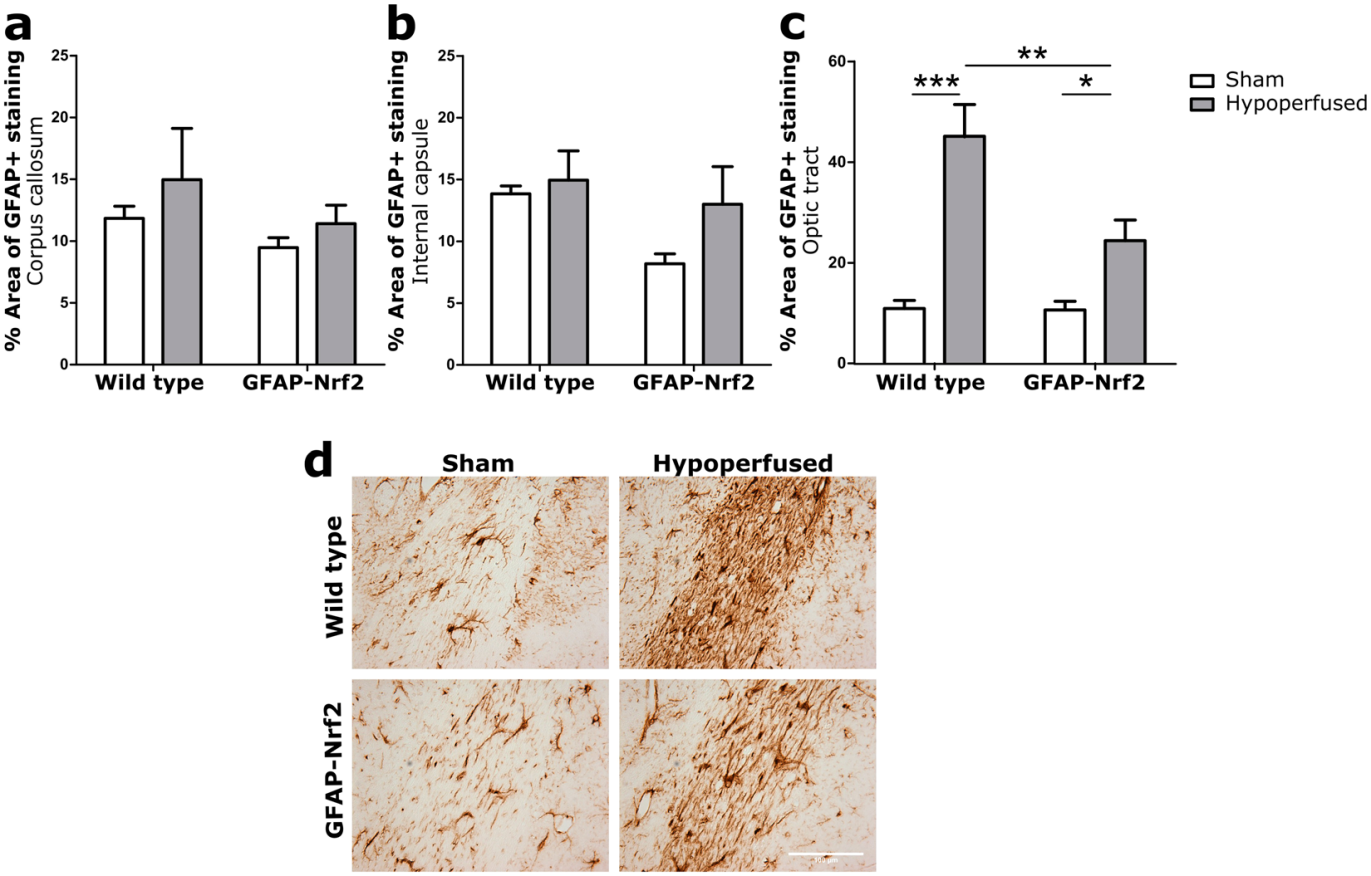
Astrogliosis in response to hypoperfusion was less severe in GFAP-Nrf2 animals. (**a**) % Area of GFAP+ staining in the corpus callosum and (**b**) internal capsule was unchanged following cerebral hypoperfusion. (**c**) % Area of GFAP+ staining in the optic tract was significantly increased by cerebral hypoperfusion (F_(1,32)_ = 38.87, p < 0.0001), with a significant effect of genotype (F_(1,32)_ = 7.45, p = 0.01). (**d**) Representative images of GFAP+ staining in the optic tract. Scale bar 100 µm. Mean ± SEM. Two-way ANOVA with Bonferroni adjustment for post hoc analysis. *p < 0.05, **p < 0.01, ***p < 0.001. n = 8–10 per group.

GFAP + astrocytes were significantly affected by hypoperfusion in the optic tract (F_(1,32)_ = 38.87, p < 0.0001) (Fig. 5c) but, similar to Iba1 expression and white matter integrity, not in the corpus callosum or the internal capsule (F_(1,32)_ = 1.43, p = 0.24, F_(1,32)_ = 2.01, p = 0.17 respectively) (Fig. 5a,b). There was also no effect of genotype in these regions (F_(1,32)_ = 1.95, p = 0.17, F_(1,32)_ = 3.34, p = 0.08 respectively). However, there was an effect of genotype in the optic tract (F_(1,32)_ = 7.45, p = 0.01) and a significant interaction between hypoperfusion and genotype (F_(1,32)_ = 7.05, p = 0.01). Post hoc tests identified significantly higher GFAP+ percentage are in hypoperfused wild type (p < 0.001) and GFAP-Nrf2 groups (p = 0.01) but also a significant reduction in the GFAP-Nrf2 hypoperfused group compared to the hypoperfused wild types (p = 0.001) (Fig. 5c). This data indicates that astrocytic Nrf2-overexpression reduces astrogliosis induced by cerebral hypoperfusion in the optic tract.

### Anti-oxidant-related genes *Slc7a11* and *Gclm* are increased by Nrf2-overexpression

Relative gene expression of *Nrf2*, *Scl7a11* (xCT; encoding the glutamate/cystine antiporter) and *Gclm*, (glutamate-cysteine ligase enzyme subunit), two Nrf2-regulated genes involved in glutathione synthesis were measured in a whole brain slice corresponding to the level of pathological assessment using qPCR and compared across groups.

**Figure 6:**
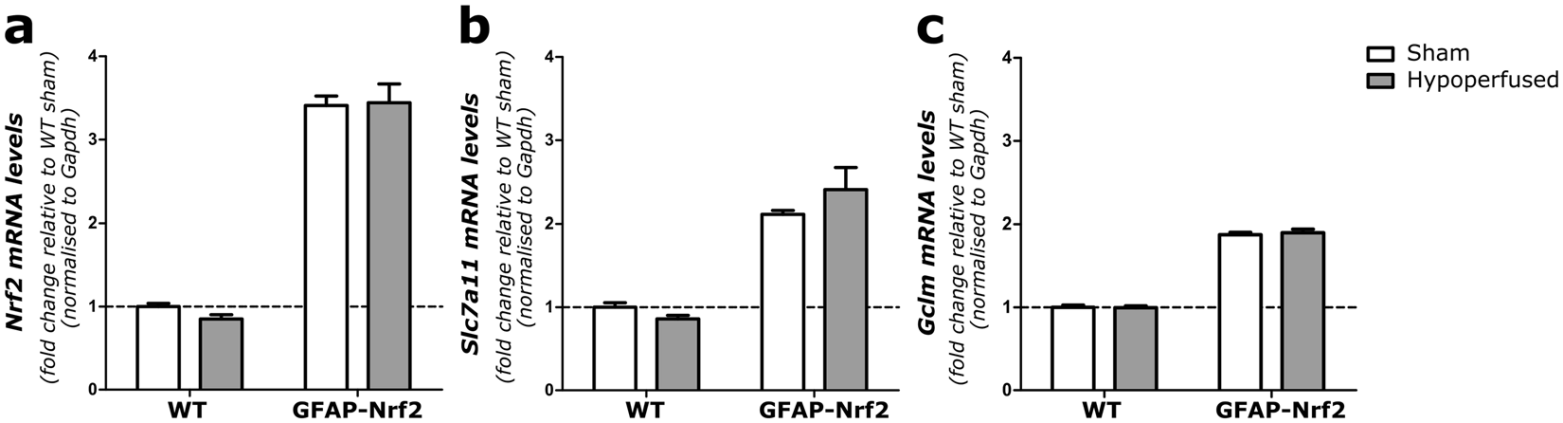
Nrf2, Slc7a11 and Gclm relative gene expression is higher in GFAP-Nrf2 animals with no effect of hypoperfusion. (**a**) *Nrf2*, (**b**) *Slc7a11* and **(c)** *Gclm* expression was significantly higher in GFAP-Nrf2 animals (F_(1,32)_ = 298, p < 0.0001, F_(1,32)_ = 74.05, p < 0.0001, F_(1,32)_ = 737.5, p < 0.0001; respectively), but this was not altered by hypoperfusion. Gene expression was normalised to *Gapdh* and expressed relative to WT sham controls. Dashed line indicates average WT sham level. Mean ± SEM. Two-way ANOVA. n = 8–10 per group.

There was a significant 3.5-fold increase in *Nrf2* mRNA expression in GFAP-Nrf2 animals compared to wild type (F_(1,32)_ = 298, p < 0.0001), with no effect of hypoperfusion (F_(1,32)_ = 0.16, p = 0.69) (Fig. 6a). In addition, these changes were accompanied by a ~2-fold increase in *Slc7a11* and *Gclm* in GFAP-Nrf2 mice (F_(1,32)_ = 74.05, p < 0.0001, F_(1,32)_ = 737.5, p < 0.0001 respectively), again, with no effect of hypoperfusion (F_(1,32)_ = 0.26, p = 0.61, F_(1,32)_ = 0.08, p = 0.79). These results may indicate a greater anti-oxidant capacity of GFAP-Nrf2 animals compared to wild types. Since there was no effect of hypoperfusion on the expression levels of these genes we considered whether subtle alterations in gene expression in predominantly white matter may be masked by measurements of gene expression in total brain homogenates. We therefore next investigated gene expression in white matter enriched samples.

### Cerebral hypoperfusion induces pro-inflammatory gene expression in the optic tract and Nrf2-overexpression reduces the expression of complement component 4

**Figure 7:**
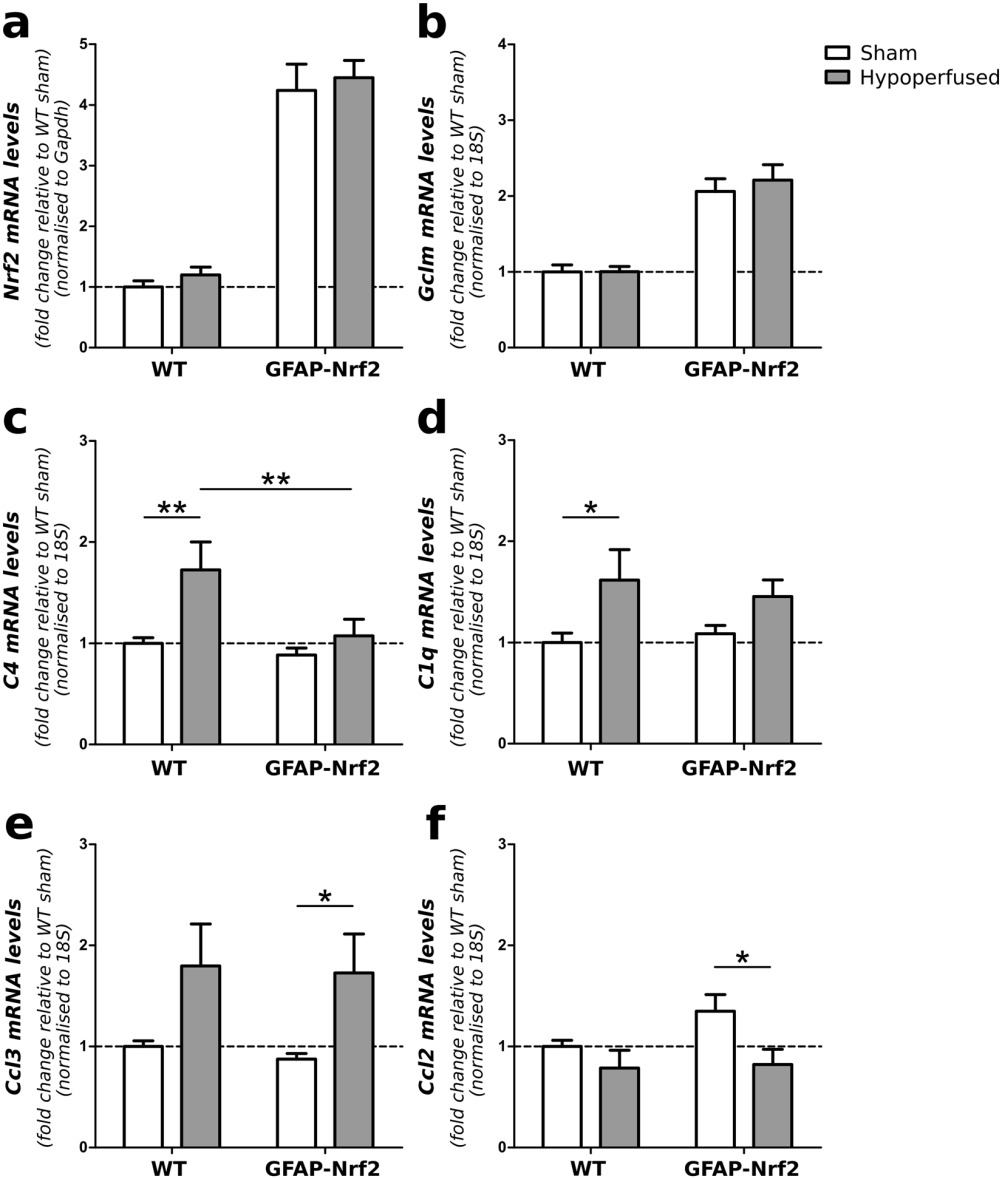
Cerebral hypoperfusion induces pro-inflammatory gene expression in the optic tract and Nrf2-overexpression reduces the expression of complement component 4. (**a**) *Nrf2* and (**b**) *Gclm* expression in the optic tract was significantly higher in GFAP-Nrf2 animals (F_(1,32)_ = 116.4, p < 0.0001, F_(1,31)_ = 49.83, p < 0.0001 respectively), but was not altered by hypoperfusion. (**c**) *C4* expression in the optic tract was significantly increased by hypoperfusion (F_(1,32)_ = 8.07, p = 0.008) with a significant effect of genotype (F_(1,32)_ = 5.66, p = 0.02). (**d**) Hypoperfusion significantly increased *C1q* and (**e**) *Ccl3* expression in the optic tract (F_(1,32)_ = 7.92, p = 0.008, F_(1,29)_ = 8.68, p = 0.006; respectively), but reduced the expression of *Ccl2* (F_(1,31)_ = 6.09, p = 0.02). There was no effect of genotype on the expression of *C1q*, *Ccl3* or *Ccl2*. Gene expression was normalised to *Gapdh* or *18S* and expressed relative to WT sham controls. Dashed line indicates average WT sham level. Mean ± SEM. Two-way ANOVA with Bonferroni adjustment for post hoc analysis. *p < 0.05, **p < 0.01, n = 8–10 per group.

Alterations in gene expression were investigated in optic tract enriched samples since we had previously determined glial alterations in this region. *Nrf2* expression in the optic tract was similar to that of the whole brain sample with an increase in GFAP-Nrf2 animals compared to wild type (F_(1,32)_ = 116.4, p < 0.0001), and no further effect of hypoperfusion (F_(1,32)_ = 0.47, p = 0.5) (Fig. 7a). To further investigate the involvement of the glutathione anti-oxidant system we measured *Gclm* expression in the optic tract enriched samples, but similarly found an effect of genotype only (F_(1,31)_ = 49.83, p < 0.0001) and no effect of hypoperfusion (F_(1,31)_ = 0.22, p = 0.65) (Fig. 7b). Collectively our results suggest that anti-oxidant gene alterations cannot explain the white matter protection conferred in hypoperfused GFAP-Nrf2 mice at this time. As an alternative, we explored the inflammatory milieu beyond the cellular responses as an alternative mechanism whereby Nrf2 may confer protection following hypoperfusion. We measured gene expression of complement component 4 and 1q (*C4, C1q)*; initiatiors of the complement system which play an important role in the innate immune response^43^. In addition, we measured gene expression of two chemokines; *Ccl3 (Mip-1α)* and *Ccl2 (Mcp-1)*, which have previously been shown to be upregulated at the protein level following severe hypoperfusion^18^.

Hypoperfusion significantly increased the expression of *C4, C1q*, and *Ccl3* expression (F_(1,32)_ = 8.07, p = 0.008, F_(1,32)_ = 7.92, p = 0.008, F_(1,29)_ = 8.68, p = 0.006; respectively) (Fig. 7c–e), whereas *Ccl2* expression was reduced by hypoperfusion (F_(1,31)_ = 6.09, p = 0.02). *C4* expression was also significantly different in GFAP-Nrf2 animals (F_(1,32)_ = 5.66, p = 0.02) and post hoc analysis found significant upregulation in wild type hypoperfused (p = 0.005) but not GFAP-Nrf2 hypoperfused animals (p = 0.39), and further that *C4* expression was significantly lower in GFAP-Nrf2 compared to wild type hypoperfused animals (p = 0.007). *C1q* expression followed a similar pattern, however there was no overall genotype effect (F_(1,32)_ = 0.05, p = 0.83). Post hoc tests found a significant increase of *C1q* in hypoperfused wild types compared to shams (p = 0.02), but found no significant difference between GFAP-Nrf2 groups (p = 0.13). No effect of genotype was detected on *Ccl3* and *Ccl2* expression (F_(1,29)_ = 0.12, p = 0.73, F_(1,31)_ = 1.62, p = 0.21; respectively), and post hoc analysis identified significant differences between GFAP-Nrf2 groups (p = 0.03, p = 0.01; respectively) but not between wild type groups (p = 0.07, p = 0.35; respectively). Together the data indicates that cerebral hypoperfusion increases the expression of pro-inflammatory genes and that Nrf2-overexpression in astrocytes may protect white matter integrity and behavioural performance by selectively suppressing aspects of this inflammatory response.

## Discussion

The present study demonstrates that astrocytic overexpression of Nrf2 exerts beneficial effects in a mouse model of chronic cerebral hypoperfusion and alleviates loss of white matter integrity and astrogliosis in the optic tract, in parallel with improved functional impairment. These protective effects appear to be mediated via repression of specific inflammatory genes.

In agreement with previous studies, we show that bilateral carotid artery stenosis (BCAS) induces mild, sustained cerebral hypoperfusion in wild type and Nrf2-overexpressing mice^15^. The extent of CBF reduction (~30–40% at 24 hours) and gradual amelioration over 6 weeks is comparable to previously published studies^9,44,45^. Notably, we found no difference in the extent of CBF reduction between wild type and GFAP-Nrf2 mice at 24 hours and 6 weeks post-BCAS. We may have expected differences between the groups as the absence of Nrf2 has previously been shown to have vascular related effects acting to impair angiogenic capacity *in vitro*^46–48^ and inhibit the upregulation of vascular endothelial growth factor (VEGF) in an *in vivo* model of venous hypertension^49^. We find increased VEGF expression and other angiogenic genes at 3 days following hypoperfusion^12^, and promoting vascular remodelling is currently explored in models of hypoperfusion to establish its potential in the treatment of vascular dementia, as discussed in^50^. The current study found that astrocytic Nrf2-overexpression does not alter CBF responses following BCAS surgery, which therefore allowed us to investigate the effects of Nrf2 without confounding flow-related differences.

Consistent with previous studies, we show that hypoperfusion induces impairments in spatial behaviour as measured by the radial arm maze in wild type mice following bilateral carotid artery stenosis^11,13^. Wild type and GFAP-Nrf2 sham mice were able to learn the task with increasing trial duration but the hypoperfused mice in both cohorts were impaired compared to their controls. It was noted that the baseline learning pattern was different in the wild type and GFAP-Nrf2 mice. To the best of our knowledge no previously published studies using this transgenic mouse line have investigated cognitive changes, and we found no reports of altered cognition at baseline in models of Nrf2-overexpression/activation. One explanation may be a disruption in redox balance. Reactive oxygen species (ROS), albeit detrimental in excess, are cell signalling molecules^51^ important for synaptic plasticity^52,53^, and it is possible that Nrf2-overexpression affects spatial behaviour by excessive ROS depletion. Despite this different baseline learning pattern, GFAP-Nrf2 sham mice successfully learned the task and the extent of impairment in spatial working memory in GFAP-Nrf2 hypoperfused mice was significantly less pronounced as compared to wild type mice. Thus the data supports our hypothesis that increased expression of Nrf2 may protect against hypoperfusion induced functional impairment.

Since the functional impairment was less pronounced in GFAP-Nrf2 mice this suggested that there may be less extensive pathological alterations. Previous studies, including ours, indicated that white matter is particularly vulnerable to cerebral hypoperfusion^9–11,54^. There is progressive axon-glial disruption,^12,15^, myelin disruption^10,11,14^ and reduced corpus callosal volume following cerebral hypoperfusion^55^. The current study agrees with previous findings of hypoperfusion-induced loss of white matter integrity, however only in the optic tract where the extent of pathology is more severe than previously reported. The main difference between this study and former is the background strain on which the animals were bred; FVB/C57Bl/6J F1 compared to pure C57Bl/6J. There is a possibility that the optic tract in FVB/C57Bl/6J F1 mice is more vulnerable to hypoperfusion than C57Bl/6J mice due to genetic differences. It is also reported that the cerebral vasculature of different strains is differently organised and may therefore present with different spatial distribution and severity of hypoperfusion^56^. The heightened vulnerability of the optic tract may also account for the impaired behaviour in the radial arm maze in the hypoperfused mice. Pure FVB/N mice are known to be visually impaired from an early age due to a mutation causing retinal degeneration^57^. However, it is unlikely that the mice were visually impaired since our FVB/C57 first generation cross successfully learned the visual-dependent radial arm maze task and we observed no altered behaviour indicating visual impairment in any of the mice. However, we cannot discount that disruption of the optic tract in hypoperfused mice resulted in impaired visual acuity and impaired performance on the radial arm maze task.

Protection against white matter damage using Nrf2-activating compound DMF has been reported in a mouse model of experimental autoimmune encephalitis (EAE)^30^ and severe hypoperfusion^18^, both of which present with substantial white matter disruption. The subtle pathology observed in this study, compared to above and previously published 1 month hypoperfusion studies^10–12,15^, may have limited the ability to investigate the modulation of pathology with Nrf2-overexpression completely. Nonetheless, despite these confounds, there was an effect of GFAP-Nrf2 overexpression on the extent of white matter pathology in the optic tract and notably these mice also exhibited improved behavioural abilities. Thus the data indicate that astrocyte specific Nrf2-overexpression may alleviate behavioural impairments by preventing loss of white matter integrity in the optic tract.

We have shown previously in the hypoperfusion model that microgliosis precedes significant increases in reactive astrocytes, and associations between microgliosis and impaired white matter function suggests that microglia are important contributors to the disease process^17,18^. Other groups have shown associations between astrogliosis and impaired white matter integrity^58^ and spatial working memory following cerebral hypoperfusion^59^, suggestedly through activation of the pro-inflammatory transcription factor NF-κB^58^ and reduced astroglial glutamate uptake^59^. In the current study, both micro- and astrogliosis were increased, however only in the optic tract of hypoperfused mice. The regional discrepancy between this study and previous may be attributed to the background strain, which as previously mentioned, may respond to hypoperfusion differently due to do variances in cerebrovascular arrangement^56^. Overexpression of Nrf2 reduced the extent of astrogliosis, but did not have an effect on microgliosis after hypoperfusion. This was somewhat unexpected as GFAP-Nrf2 mice have previously been shown to reduce both astro- and microgliosis in models of Parkinson’s Disease^37,38^, and Nrf2-deficiency induced robust microgliosis in a model of EAE^29^. There is conflicting evidence of the effect of Nrf2 on gliosis in other models, for example in the transgenic Alzheimer’s disease mouse model APP/PS1. One study found that only astrogliosis was reduced by Nrf2^60^, and another found that both astro- and microgliosis were affected^61^, however both were associated with improved cognition. The differences may be accounted for by different means of Nrf2-activation; lentiviral-Nrf2 hippocampal injection^60^, oral administration of the Nrf2-activating compound methysticin^61^ or transgenic overexpression of Nrf2 in astrocytes^37,38^. Our contrasting results from previous studies in GFAP-Nrf2 mice may be explained by the different experimental models, and perhaps particularly by the comparably more subtle pathology. Importantly this study demonstrates that increased Nrf2 expression in astrocytes alone can have downstream beneficial effects on optic tract white matter integrity and behaviour. Together, these result further support the therapeutic potential of activating the Nrf2-pathway in cerebrovascular and neurodegenerative disease. A plethora of pharmacological compounds capable of activating the Nrf2-pathway are currently under investigation (reviewed in^62,63^).

To probe mechanisms by which Nrf2 overexpression may mediate protective effects, the levels of two glutathione-related genes; *Slc7a11* (*xCT*) and *Gclm* were measured. The glutathione system forms an important part of cellular anti-oxidant capacity^64^ and has been shown previously to be upregulated by ischaemic preconditioning in an Nrf2-dependent manner^40^, as well as in GFAP-Nrf2 mice in several models of neurodegenerative disease^35–37^. We found an increase in both *Slc7a11* and *Gclm* in GFAP-Nrf2 compared to wild type mice, however contrary to expectations there were no further increases in these genes with hypoperfusion. Given that hypoperfusion induces oxidative damage^23,65^, we expected increased anti-oxidant gene expression also in wild type hypoperfused animals indicative of Nrf2-pathway activation. Instead, anti-oxidant enzymes were determined to be increased only in GFAP-Nrf2 animals; in line with a study in a transgenic model of Parkinsons disease^37^. Our results suggest that hypoperfusion in wild type mice is insufficient to induce Nrf2 expression and activation directly, although the constitutively higher glutathione-related gene expression in the GFAP-Nrf2 animals may indicate a higher capacity of glutathione synthesis which may provide some protection to white matter following hypoperfusion. However, we are unable to conclude this without direct measures of glutathione. A model of Alexander disease found glutathione-independent protective effects of GFAP-Nrf2 expression^66^, and a proteomic study of Nrf2-overexpressing astrocytes found important roles of detoxifying and anti-inflammatory enzymes catalase, peroxiredoxin-6 and prostaglandin reductase 1^67^ which could alternatively be implicated following hypoperfusion as opposed to increased glutathione synthesis. The inability to detect anti-oxidant gene expression may be explained by the lack of sensitivity of transcriptome measures in total brain homogenates but since we determined similar effects in optic-tract enriched samples, where prominent axon-glial alterations were detected, a lack of gene alterations is unlikely due to sampling. An alternate explanation is that the protective effect seen in the study is mediated by modulation of the inflammatory environment rather than by anti-oxidant mechanisms. We investigated this by further qPCR analysis of optic tract-enriched samples of a panel of pro-inflammatory genes; *C4* and *C1q*; complement component 4 and 1q, and *Ccl3 (MIP-1α)* and *Ccl2 (MCP-1);* chemokine (C-C motif) ligand 3 and 2. The complement system is an important part of the innate immune system, aiding early responses to infection or injury. It contributes to the adaptive immune system by interacting with antigen/antibody complexes eliciting a cascade of proteolytic events which ultimately boost phagocytosis and inflammation by the recruitment of more inflammatory cells^43^. *Ccl3/Ccl2* encode pro-inflammatory chemokines involved in the recruitment of microglia and astrocytes as well as peripheral monocyts and macrophages^68,69^. Increased expression of complement components as well as pro-inflammatory chemokines has long been recognised as an indication of chronic inflammation for example in Alzheimer’s disease^70–72^. Our previous work found increased expression of *C4* and a putative receptor for C1q, in white matter at 72 hours post-hypoperfusion^12^ and in whole brain at 4 weeks following mild hypoperfusion in wild type mice (*unpublished obeservations*) using microarray analysis, and increased protein levels of Ccl3 and Ccl2 following severe hypoperfusion^18^. Consistent with previous work, an increase in *C4, C1q* and *Ccl3* expression following hypoperfusion was determined in wild type mice, indicative of ongoing inflammation. In Nrf2 overexpressing mice the extent of this increase in complement related genes was dampened. C1q as the initiating molecule in the classical complement pathway provides the downstream product for which C4 is a substrate^73^. Complement activation has been suggested to potentiate chronic inflammation and neurodegeneration^74,75^, and has been demonstrated to trigger neuroinflammation following traumatic brain injury^76^. Since direct inhibition of complement has been neuroprotective in mouse models of Alzheimer’s disease^77^ and cerebral ischaemia^78,79^, reduced complement activation may be a mechanism whereby Nrf2-overexpression is protective following hypoperfusion. A recently proposed hypothesis of neuroinflammation is that activated microglia release pro-inflammatory mediators (including complement C1q and C3) inducing astrocyte reactivity which in turn is detrimental to surrounding cells and tissues^74^. Our results in astrocyte-specific Nrf2-overexpressing animals find reduced astrogliosis but similar coverage of microglia as wild types, reflected also by comparable levels of expression of the chemokine *Ccl3*. If microglia are responsible for inducing astrogliosis, reduced astrogliosis in this study may result from less pro-inflammatory signalling from microglia in Nrf2-overexpressing animals. Recent evidence has demonstrated that Nrf2 not only induces the expression of cytoprotective genes, but is also able to suppress expression of pro-inflammatory genes directly^80^. Since the Nrf2-overexpression is selectively in astrocytes, another possibility is that these may also be more resistant to microglia-driven pro-inflammatory signalling as a result of increased Nrf2-signalling. In contrast to our previous work where Ccl2 protein was increased at 1 week after severe hypoperfusion,^18^, *Ccl2* expression was determined to be downregulated with mild hypoperfusion at 6 weeks. The discrepancy may be due to differences in anatomical areas sampled which may display altered pro-inflammatory responses and/or the differing extent of hypoperfusion. Overall, the differential change in inflammatory gene expression likely reflects the spatial and temporal heterogeneity of inflammatory responses to hypoperfusion.

In conclusion, these results indicate that Nrf2 overexpression in astrocytes dampens aspects of hypoperfusion-induced inflammation in the optic tract, possibly by Nrf2-driven suppression of pro-inflammatory genes or by increased anti-inflammatory gene expression, ultimately reducing optic tract astrogliosis and loss of white matter integrity paralleled by improved functional outcome. This adds support to the use of Nrf2-activators as potential treatment for cerebrovascular-related inflammation and white-matter degeneration.

## Methods

### Animals

GFAP-Nrf2.2 mice were imported from Prof. JA Johnson, University of Wisconsin, and were on an FVB background. These mice were developed as previously described in^35^ and Nrf2-overexpression in astrocytes was driven by a GFAP promoter. GFAP-Nrf2 mice were then crossed with wild type C57Bl/6J in-house. The first generation crosses including transgenic GFAP-Nrf2 and wild type littermates aged 4–5 months were utilised for the study (GFAP-Nrf2, FVB/C57Bl/6J F1). All mice used for the studies were on the same genetic background. Animals were initially group housed on a 12-hour light/dark cycle with *ad libitum* access to food and water, and assigned experimental groups by genotype then randomly assigned surgery; wild type sham (n = 8), wild type hypoperfused (n = 10), GFAP-Nrf2 sham (n = 10), GFAP-Nrf2 hypoperfused (n = 12). All mice were male. All experiments were conducted in accordance with the Animal (Scientific Procedures) Act 1986 and local ethical approval at the University of Edinburgh, and were performed under personal and project licences granted by the Home Office according to ARRIVE guidelines. Four animals tolerated surgery poorly and had to be culled. Final numbers were hence as follows; wild type sham (n = 8), wild type hypoperfused (n = 8), GFAP-Nrf2 sham (n = 10), GFAP-Nrf2 hypoperfused (n = 10). Experimenters were blind to genotype and surgery status of the mice throughout data collection and analysis.

### Bilateral carotid artery stenosis

Animals underwent bilateral carotid artery stenosis (BCAS) surgery as developed by^9^ under isoflurane anaesthesia using 0.18 mm internal diameter microcoils placed around both common carotid arteries. Sham surgery includes the entire procedure except for placement of the microcoils. All surgical procedures were conducted using aseptic techniques.

### Laser speckle imaging

Baseline measurements of cortical cerebral perfusion were acquired prior to, 24 hours and 6 weeks post-BCAS surgery using a Moor FLPI2 laser speckle contrast imager (Moor Instruments, UK). The animal was anaesthetised using isoflurane and its’ head held in position using a stereotactic frame. Body temperature was regulated using a heated pad and the skull was exposed by a midline incision and reflection of the skin of the head. Water-based gel was evenly spread on the exposed skull and a 2-min perfusion recording was acquired. The skull was then sutured and local anaesthetic applied to reduce any pain or discomfort to the animal which was then recovered in a heat regulated box.

### 8-arm radial arm maze to assess behavioural alterations

Animals were singly housed and food restricted (maintained at 85% of initial body weight) one week prior to, and throughout the radial arm maze test, to promote motivation (12-hour light/dark cycle, *ad libitum* access to water). Following the last trial animals were again provided food *ad libitum*. The radial arm maze test was commenced one month after hypoperfusion.

The radial arm maze comprises a central platform (20 cm in diameter) surrounded by 8 arms (47 cm long by 7 cm wide with 20 cm Plexiglas walls). Each arm has a 2-cm deep plastic well for placement of a sugar pellet and all arms can be isolated from the central platform by Plexiglas doors (remotely controlled using Any-Maze software, Stoelting, UK). Large visual cues were placed on each of the four walls surrounding the maze, and a camera mounted on the ceiling was used for data acquisition (Any-Maze software, Stoelting, UK).

Pre-training consisted of one 5 min trial of free exploration with sugar pellets scattered at random, and one where each animal was allowed to walk down each arm from the central platform to retrieve sugar pellets from the plastic cups.

The training was carried out for 16 consecutive days (1 trial/day). Each arm was baited with a sugar pellet and the animal was placed in the central platform at the start of the trial. The animal was confined to the central platform for 5 seconds between each arm choice and the trial finished when the animal had retrieved all 8 pellets or when 25 minutes had elapsed. The number of revisiting errors (visits into unbaited arms) during each trial were recorded and analysed as a measure of behaviour.

Animals that explored less than 75% of the maze during 2 of the first 4 trials were excluded from analysis due to lack of motivation resulting in skewed learning profile. That resulted in the final numbers for the behavioural analysis; wild type sham (n = 5), wild type hypoperfused (n = 6) GFAP-Nrf2 sham (n = 10), GFAP-Nrf2 hypoperfused (n = 10).

### Tissue processing

Following the behavioural testing, animals were sacrificed under deep anaesthesia by transcardiac perfusion and hemi brains were snap frozen in liquid nitrogen or post-fixed in 4% paraformaldehyde for 24 hours and further processed for paraffin embedding. 6 µm thick coronal tissue sections were collected at −1.70 mm posterior of bregma according to^81^ using a rotary microtome (Leica Biosystems, Germany). The corresponding level of the frozen hemi brain was used for RNA extraction and qPCR analysis. In some experiments the optic tract was identified and isolated to produce preparations enriched in optic tract for qPCR analysis.

### Immunohistochemistry

Standard laboratory procedures were utilised for immunostaining. Sections were deparaffinised and endogenous peroxidase quenched in 3% H_2_O_2_ in methanol and antigen retrieval was carried out when required (Iba1 and GFAP) in 10 mM citric acid buffer (pH 6.0) at 95 °C for 10 min. Sections were blocked with 10% normal serum and 0.5% bovine albumin serum before overnight primary antibody incubation at 4 °C. Biotinylated secondary antibodies were incubated for 1 hour at room temperature and then further amplified; 1 hour at room temperature in Vector ABC Elite Kit (Vector Labs, UK), before visualisation of peroxidase activity using 3,3′ diaminobenzidine tetrahydrochloride (DAB, Vector Labs, UK). Primary antibodies and concentration used were as follows; Iba1 rabbit polyclonal Menarini, cat no. MP-290, 1:1000; GFAP rat monoclonal Life Technologies, cat no. 13-0300, 1:1000; MAG mouse monoclonal Abcam, cat no. ab89780, 1:15,000. Images were acquired using an Olympus BX51 microscope (x20, Olympus, UK) and analysed using ImageJ software (v1.46, NIH, Bethesda, MD, USA). The density of MAG+, Iba1+ and GFAP+ immunostaining was quantified as percentage area. The background was subtracted and a global manual threshold applied. All stains were evaluated in the corpus callosum, the internal capsule and the optic tract which were manually delineated by the experimenter.

### RNA extraction, reverse transcription-PCR and quantitative (q)-PCR

RNA was extracted using the QIAGEN RNeasy Lipid Tissue Mini Kit according to manufacturer’s instruction. Briefly, <100 mg fresh frozen tissue was homogenised in 1 ml QIAzol® lysis reagent using the Qiagen automated tissue lyser system and metal beads. The homogenate was transferred to fresh RNase/DNase free tubes and incubated for a couple of minutes at room temperature with 200 µl chloroform. The upper aqueous phase was collected following 15 min centrifugation at 4 °C (12,000 × *g*), and RNA was subsequently purified in mini spin columns and washed with a series of buffers before it was eluted in RNase free water. RNase-free DNase I (Thermo Scientific) was used to remove genomic DNA according to manufacturer’s instruction. cDNA was synthesised from 0.1–1 µg RNA using the Roche Transcriptor First Strand cDNA Synthesis Kit, according to manufacturer’s instruction. Briefly, RNA was added to reverse transcriptase (RT) reaction mix and cycled through 10 min 25 °C; 30 min 55 °C; 5 min 85 °C. NoRT control was run alongside and cDNA was diluted to the equivalent of 3 ng initial RNA per 15 µl qPCR reaction. The CFX96 Real-Time PCR Machine (Bio Rad) was used with the DyNAmo ColorFlash SYBR Green qPCR kit according to manufacturer’s instructions (Thermo Scientific). cDNA template was mixed with SYBR green master mix, water and forward and reverse primer (200 nM each final concentration). Samples were run in duplicates alongside no template and no RT negative controls. Primers were validated to confirm efficiency prior to use and sequences used are as follows: *Gapdh*-F: 5′-GGGTGTGAACCACGAGAAAT-3′, *Gapdh*-R: 5′-CCTTCCACAATGCCAAAGTT-3′, *18S*-F: 5′-CCCAGTAAGTGCGGGTCAT-3′, *18S*-R: 5′-CCGAGGGCCTCACTAAACC-3′, *Nrf2*-F: 5′-CAGCTCAAGGGCACAGTGC-3′, *Nrf2*-R: 5′-GTGGCCCAAGTCTTGCTCC-3′, *Slc7a11*-F: 5′-ATACTCCAGAACACGGGCAG-3′, *Slc7a11*-R: 5′-AGTTCCACCCAGACTCGAAC-3′, *Gclm*-F: 5′-GCACAGCGAGGAGCTTC-3′, *Gclm*-R: 5′-GAGCATGCCATGTCAACTG-3′, *C4*-F: 5′-ACAACAAGGGAGACCCCCAG-3′, *C4*-R: 5′-GCTCAGAGAGCCAGAGTCCTA-3′, *C1q*-F: 5′-CAAGGACTGAAGGGCGTGAA-3′, *C1q*-R: 5′-CAAGCGTCATTGGGTTCTGC-3′, *Ccl3*-F: 5′-GCCAGGTGTCATTTTCCTGACT-3′, *Ccl3*-R: 5′-TCAGGCATTCAGTTCCAGGTC-3′, *Ccl2*-F: 5′-TCCCAAAGAAGCTGTAGTTTTTGTC-3′, *Ccl2*-R: 5′-CCCATTCCTTCTTGGGGTCA-3′.

The qPCR cycling programme was 10 min at 95 °C; 40 cycles of 30 s at 95 °C, 30 s at 65 °C (/30 s at 62.5 °C for *C1q* and *Ccl2* experiment/40 s at 60 °C for *Gclm* experiment, 62.5 °C) with detection of fluorescence, 30 s at 72 °C; 1 cycle (for dissociation curve) of 1 min at 95 °C and 30 s at 55 °C with a ramp up to 30 s at 95 °C with continuous detection of fluorescence The *C4* experiment was run at 7 min at 95 °C initially and then 40 cycles of 10 s at 95 °C, 30 s at 65 °C (/30 s at 60 °C for *Ccl3* experiment) with detection of fluorescence followed by the dissociation curve. Data was normalised to *Gapdh* or *18S* expression as reference and expressed as fold change of wild type sham expression.

Due to minute volumes of optic tract enriched RNA samples, more variation was present in these qPCR experiements. Criteria were therefore defined to exclude samples from qPCR analysis if housekeeper gene was detected >3Ct away from the mean or if gene of interest expression was 1.5 interquartile ranges less than the first quartile, or 1.5 interquartile ranges more than the third quartile for each group mean. The *Ccl3* experiment therefore had three samples exluded, and *Gclm* and *Ccl2* experiments one sample each excluded from analysis. Final numbers for these experiments were hence as follows; *Ccl3* -wild type sham (n = 7), wild type hypoperfused (n = 7) GFAP-Nrf2 sham (n = 10), GFAP-Nrf2 hypoperfused (n = 9), *Gclm* - wild type sham (n = 7), wild type hypoperfused (n = 8) GFAP-Nrf2 sham (n = 10), GFAP-Nrf2 hypoperfused (n = 10) and *Ccl2* - wild type sham (n = 8), wild type hypoperfused (n = 7) GFAP-Nrf2 sham (n = 10), GFAP-Nrf2 hypoperfused (n = 10). All other qPCR experiments included all samples.

### Statistical analysis

Statistical analysis was performed using SPSS (v22, IBM Corp.) or Graphpad Prism (v5, GraphPad Software Inc, La Jolla, USA). Data are presented as mean ± SEM. Normally distributed data was analysed by analysis of variance (ANOVA). Repeated measure ANOVA was used to analyse cerebral blood flow and revisiting errors over time/trials and 2-way ANOVA was performed to test for hypoperfusion/genotype effects of immunohistochemistry and qPCR data. Bonferroni adjustment was used for post hoc analysis. Significance was determined at p < 0.05.

## Electronic supplementary material

Supplementary Information

## Data Availability

The data sets generated during the current study are available from the corresponding authors upon reasonable request.
