# Supplementary material for: Astrocyte-specific overexpression of Nrf2 protects against optic tract damage and behavioural alterations in a mouse model of cerebral hypoperfusion
Source: Sci Rep. 2018 Aug 22;8:12552. doi: 10.1038/s41598-018-30675-4 (PMC6105641; doi:10.1038/s41598-018-30675-4)
Supplement: Supplementary file 1 — Supplementary Information [file 41598_2018_30675_MOESM1_ESM.docx]

# SUPPLEMENTARY INFORMATION

# Astrocyte-specific overexpression of Nrf2 protects against optic tract damage and behavioural alterations in a mouse model of cerebral hypoperfusion

## Authors:

Emma Sigfridsson^1^, Martina Marangoni^1,2^, Jeffrey A. Johnson^3,4,5,6^, Giles E. Hardingham^1,7,8^, Jill H. Fowler^1^*, Karen Horsburgh^1^*

## Author Affiliations:

^1^Centre for Discovery Brain Sciences, University of Edinburgh, Edinburgh, UK

^2^Current address: Department of Health Sciences, University of Florence, Florence, Italy.

^3^Division of Pharmaceutical Sciences, ^4^Molecular and Environmental Toxicology Center, ^5^Center for Neuroscience and ^6^Waisman Center, University of Wisconsin. Madison, US.

^7^Edinburgh Medical School, University of Edinburgh, Edinburgh, UK.

^8^The UK Dementia Research Institute, University of Edinburgh, Edinburgh, UK.

*Joint senior authors

Correspondence and request for materials should be addressed to J.H.F (Chancellor's Building, 49 Little France Crescent, Edinburgh, EH16 4SB, UK. Email: [jill.fowler@ed.ac.uk](mailto:jill.fowler@ed.ac.uk)) or K.H. (Chancellor's Building, 49 Little France Crescent, Edinburgh, EH16 4SB, UK. Email: [karen.horsburgh@ed.ac.uk](mailto:karen.horsburgh@ed.ac.uk))

**
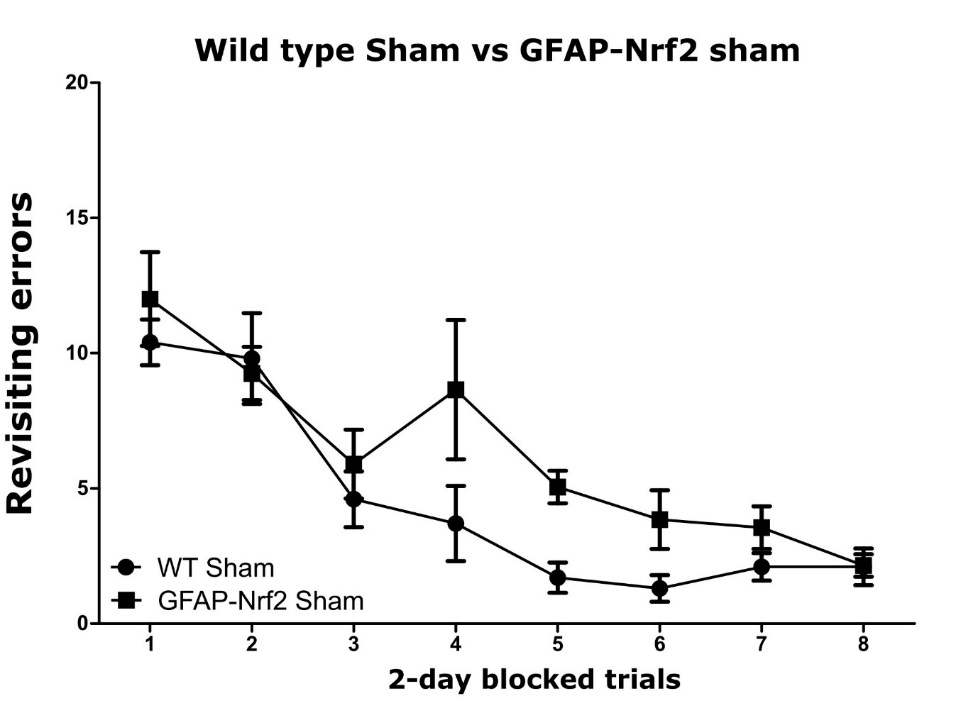
Supplementary Figure 1. Sham wild type and GFAP-Nrf2 transgenics had different learning pattern.** The learning curve for WT sham and GFAP-Nrf2 sham animals followed different patterns, however there was no statistical significance between the groups (F­_1,13)_=2.73, p=0.12). Mean ± SEM. WT sham n=5, GFAP-Nrf2 sham n=10.
